# Promotion of Disposable Electronic Cigarette Flavors and Topics on Twitter

**DOI:** 10.3390/ijerph17249221

**Published:** 2020-12-10

**Authors:** Li Sun, Chunliang Tao, Zidian Xie, Dongmei Li

**Affiliations:** 1 Goergen Institute for Data Science, University of Rochester, Rochester, NY 14647, USA; lsun12@u.rochester.edu; 2 Department of Electrical and Computer Engineering, University of Rochester, Rochester, NY 14627, USA; ctao4@u.rochester.edu; 3 Department of Clinical and Translational Research, University of Rochester Medical Center, Rochester, NY 14642, USA

**Keywords:** disposable e-cigarettes, flavors, Twitter

## Abstract

Disposable electronic cigarettes (e-cigarettes) became popular among youth after the Food and Drug Administration (FDA) implemented an enforcement policy to restrict the sale of cartridge-based flavored e-cigarettes starting from February 2020 in the United States (US). We aimed to examine the flavors and topics related to disposable e-cigarettes on Twitter. The Twitter dataset, which includes 1489 tweets, was collected by the Tweepy streamapplication programming interface (API) using a keyword query from March to September 2020. The disposable e-cigarette flavors were curated from both online stores and collected tweets. Topics related to disposable e-cigarettes on Twitter were manually coded. Distributions of topics were compared between tweets from the US and tweets from non-US countries. The temporal analysis results showed a slight increase in the number of discussions over the study period. Strawberry, mango, watermelon, and mint were the most popular flavors of disposable e-cigarettes mentioned on Twitter. Almost all the tweets (97.11%) were commercial tweets, which were dominated by topics related to the product and flavor promotions. The US tweets focused more on product and flavor promotions and less on price promotions compared to non-US tweets. Our results suggest that companies exploited the limitations of legislation to promote flavors on Twitter, which could undermine public health and young people’s finances if they get hooked on addictive products.

## 1. Introduction

A disposable electronic cigarette (e-cigarette) is a type of electronic nicotine delivery system (ENDS), which is designed to be thrown away once it runs out of charge or e-liquid [1]. Disposable e-cigarettes are different from cartridge-based e-cigarettes which consist of, include, or involve a cartridge or pod that holds liquid to be aerosolized for users to consume; the cartridge or the cartomizer can be refilled with e-liquid with/without other additives such as THC (tetrahydrocannabinol) for future use [1]. The cartridge-based e-cigarettes such as JUUL rapidly gained popularity in youth in recent years. The 2019 National Youth Tobacco Survey (NYTS) indicated that 27.5% of high-school students and 10.5% of middle-school students used e-cigarettes [2]. Increasing research evidence on e-cigarettes has indicated their associations with nicotine addiction, respiratory diseases, cardiovascular diseases, cognitive problems, and possibly cancer [3]. The ourbreak of e-cigarette or vaping use-associated lung injury (EVALI) has led to a total of 2807 hospitalized cases or deaths in the United States (US) as of 18 February 2020 [4].

To prevent the epidemic of electronic cigarette (e-cigarette) use in youth, the US Food and Drug Administration (FDA) announced the e-cigarette flavor enforcement policy on 2 January 2020 and implemented it on 6 February 2020, to prohibit the sale and distribution of cartridge-based flavored e-cigarette products (except for tobacco and menthol flavors) in the US [1]. However, disposable e-cigarette devices were not restricted by the FDA flavor enforcement policy, and they quickly became popular among the youth after the FDA flavor enforcement policy. Providing similar flavors (such as fruit flavors) to JUUL (the most popular e-cigarette products in youth before the announcement of the FDA flavor enforcement policy), disposable e-cigarettes rapidly became popular in youth due to their sleeky design, cheap price, attractive packaging, and convenience for use (no filling, no charging) [2,3]. The 2020 National Youth Tobacco Survey data showed that the use of disposable e-cigarettes significantly increased from 2.4% in 2019 to 26.5% in 2020 among high-school current e-cigarette users and from 3.3% in 2019 to 15.2% in 2020 among middle-school current e-cigarette users [5].

Social media platforms are commonly used by companies, factories, and stores to promote their products, as well as by social media users to share their opinions and perceptions [6]. Twitter, one of the most popular social media platforms, has been widely used by tobacco researchers to examine tobacco product promotions and public perceptions of tobacco products. For example, Twitter was used as a data source to investigate the social influence of e-cigarettes [7]. E-cigarette-related tweets were categorized into informational and advertising categories, and a general sentiment analysis was conducted for those tweets [8]. Twitter was used to examine e-cigarette users’ attitudes toward each flavor category on Twitter through sentiment analysis [9]. Moreover, promotion strategies used by e-cigarette merchants were also explored using Twitter data [10].

With the emergence of disposable e-cigarette use in youth, it is important to characterize the popular flavors in disposable e-cigarettes and examine their promotion methods and distributions, as well as user perceptions, through social media. In this study, we collected disposable e-cigarette flavor information from both online stores and tweets and examined their popularities. We also classified the topics of disposable e-cigarettes from commercial tweets. Different promotion techniques were further examined on the basis of regional differences. Topics from noncommercial tweets were also summarized. Given the rising popularity of disposable e-cigarette use in youth, the findings from the current study provide important information on popular flavors of disposable e-cigarettes, as well as the promotion and dissemination methods of disposable e-cigarettes using Twitter, which could provide valuable information for FDA regulatory policies.

## 2. Materials and Methods

### 2.1. Disposable E-Cigarette Flavor Data Collection, Preprocessing, and Classification

Available disposable e-cigarettes from the most popular e-cigarette online stores (such as ziipstock.com, vapesocietysupplies.com, and ejuiceconnect.com) were collected. For each online store, we manually recorded brand names, product names of each brand, and flavor names of each product for all disposable e-cigarettes. In total, 112 brand names with 146 products were collected (Table S1, Supplementary Materials). We tokenized each flavor name as many flavor names were the combination of additives and basic flavors. Words with no flavor meanings were filtered out, and repetitive flavors were deleted within a brand.

### 2.2. Twitter Data Collection and Preprocessing

Disposable e-cigarette-related tweets (Twitter posts) in the English language were crawled from 9 March 2020–18 September 2020 using the Tweepy stream application programming interface (API) using related keywords, including “disposablevape”, “bestvapedisposable”, “disposablepod”, “disposablevaporizer”, “disposablepen”, and “disposablevapeoem”. Repetitive tweets were deleted from the dataset. As a result, 1489 disposable e-cigarette-related tweets remained. According to the tweets’ contents and tweeters’ user names, the tweets were classified into either commercial tweets or noncommercial tweets. For example, if the tweet contained advertisements such as product promotion, price promotion, or sellers’ contacts, or the user name contained the keyword “vape”, it was classified as a commercial tweet. Otherwise, it was classified as a noncommercial tweet. On the basis of the geolocation information provided in tweets, we further divided the commercial tweets into US, non-US, and unknown groups. Two coders from the study team coded each tweet separately, and disagreements were discussed among team members to achieve final agreements.

### 2.3. Flavor Popularity Analysis

To determine different flavors’ popularities within selected tweets, the number of appearances of each flavor was counted. Specifically, we tokenized the flavor names to generate a keyword list of flavors. For example, “strawberry banana” would be “strawberry” and “banana” after tokenization. Then, we tokenized the text content of each tweet and calculated the total appearance of flavors on the basis of the keyword list of flavors.

### 2.4. Topic Analysis

A topic analysis was conducted to identify major topics discussed on disposable e-cigarettes by manually classifying the tweets into different topic categories. Two team members hand-coded the tweets into different topics on the basis of their contents. Due to the small number of noncommercial tweets (39 out of 1489), topics discussed in those tweets were simply summarized without further classification. For the commercial tweets, we further classified them as either explicit or implicit during product promotion. For example, tweets promoting product features, such as battery and flavor, were considered as explicit techniques as they offered specific items/aspects that were being sold. In contrast, tweets not necessarily mentioning specific products but still trying to promote sales in an indirect way such as building a good company reputation by reiterating its culture and constructing close bonds with customers were considered as implicit techniques. Tweets were further classified into different topics within either the explicit or the implicit promotion technique category. For example, within explicit techniques, the topics included “product promotion”, “flavor promotion”, “general advertisement”, “price promotion”, and “new arrival”. “Product promotion” referred to the promotion of disposable e-cigarette products in the tweets. “Flavor promotion” referred to the promotion of disposable e-cigarette flavors. “General advertisement” referred to the general advertisement on disposable e-cigarettes. “Price promotions” referred to promoting sales of disposable e-cigarettes. “New arrival” referred to the promotion of new disposable e-cigarette products. Within the implicit techniques, the topics included “sales contact”, “interaction”, “related information”, “catchphrase”, “unrelated wishes”, “company culture”, and “advocate disposable”. “Sales contact” referred to tweets including email or phone numbers or other contact information of sales persons. “Interaction” referred to the scenario where the tweets were trying to ask for opinions or inspire customers to tag their friends to share information. “Related information” referred to the information related to the vaping company, which included but was not limited to a newly opened store, disposable e-cigarette expos, and policies. “Catchphrases” were sentences or slogans that were easy to remember and could promote the sale. “Unrelated wishes” referred to the tweets conveying wish-related content such as “good morning” that did not directly promote the products but tried to build a close relationship with their customers by tweeting daily greetings. “Company culture” referred to tweets not mentioning products but emphasizing the company value. “Advocate disposable” referred to tweets supporting the sale and use of disposable e-cigarettes. A tweet was classified into multiple topic categories if it contained multiple topics. For instance, tweets with both product promotion and price promotion were classified into both the “product promotion” and the “flavor promotion” topics. Groups and topics were selected by two coders on the basis of recurring themes throughout the tweets. Intercoder disagreements were discussed to resolve any discrepancies. The distributions of topics on disposable e-cigarettes between the US group and non-US group were compared using a Pearson’s chi-square test at a 5% level of significance.

## 3. Results

### 3.1. Temporal Analysis

**Figure 1:**
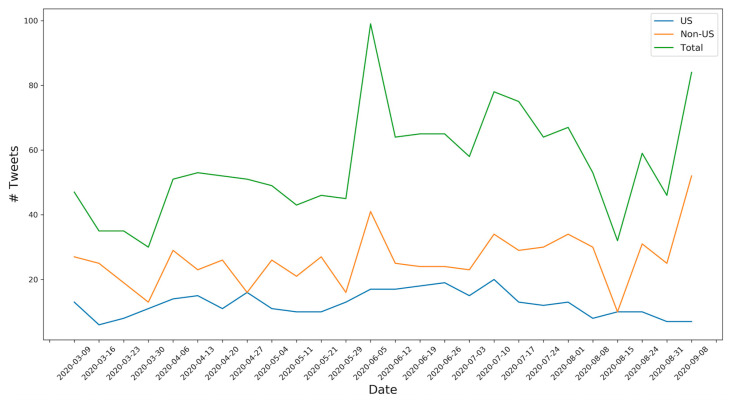
Temporal trend of the number of tweets related to disposable electronic cigarettes (e-cigarettes).

From Twitter, we collected 1489 tweets related to disposable e-cigarettes, which included 329 tweets from the US, 685 tweets from non-US countries, and 475 tweets with unknown geolocation. The number of tweets was summarized over seven days to observe the temporal trend of discussions on disposable e-cigarettes over the study period. As shown in Figure 1, there was a slightly increasing trend in the number of tweets related to disposable e-cigarettes over the study time period. While the number of tweets was steady in the US group, the number of tweets in the non-US group showed some fluctuations. For example, a peak and a trough were observed in the non-US group in the week of 5 June 2020 and the week of 15 August 2020.

### 3.2. Prevalence of Flavors in Disposable E-Cigarettes

With the increasing popularity of disposable e-cigarettes, it is important to examine which flavors are available on the market, especially online, and which flavors are popular on social media (such as Twitter). Table 1 shows the top nine flavors collected from disposable e-cigarettes online stores and disposable e-cigarettes related tweets. We found that “mango”, “blueberry”, and “strawberry” are the most popular flavors available in online stores. In contrast, “strawberry”, “mango”, and “watermelon” are the most popular flavors mentioned on Twitter. In addition, we found “ice” as the most frequent adjective for flavors in both online stores and tweets. “Ice”, “mint”, and “menthol” share the similar characteristics of providing a cooling sensation for the users. Other than these three flavors and the beverage flavors, all other flavors in the top flavor lists were fruit flavors. Mango and blueberry were the two most popular fruit flavors in online stores, while strawberry was the most popular fruit flavor on Twitter.

### 3.3. Topics Discussed on Twitter

Due to the small number of noncommercial tweets, the topic analysis was only conducted on commercial tweets. The noncommercial tweets generally showed a positive perception of disposable e-cigarette flavors and the vaping cloud generated from the disposable e-cigarettes. Table 2 shows topics identified from all commercial tweets related to disposable e-cigarettes. Classifying the commercial tweets into different topics allowed us to understand how disposable e-cigarettes were promoted on Twitter. Within the explicit techniques group, “product promotion” was the most frequent topic (634 out of 1446 tweets, 43.81%), followed by “flavor promotion” (15.21%), “price promotion” (8.09%), “general advertisement” (7.33%), and “new arrival” (5.39%). Within the implicit techniques group, the top topics were “sales contact”, “interaction”, “related information”, and “catchphrase”. Explicit techniques were more frequently used than implicit techniques in commercial tweets to promote the sale of disposable e-cigarettes on Twitter. Tweets not falling into any of these topics, where most of them did not convey any informative information, were categorized as “unsorted” in the “others” group.

Among all commercial tweets, there were 325 tweets from the US group and 680 tweets from the non-US group. For both US and non-US groups, “product promotion” was the most frequently used explicit technique (171 out of 325 tweets from the US; 298 out of 680 tweets from non-US countries) on Twitter. Among implicit techniques, “interaction” was the most frequently used technique for both the US and the non-US groups (17 out of 325 tweets from the US; 91 out of 680 tweets from non-US countries). Results from Pearson’s chi-square test indicated a difference in topic distributions between the US group and the non-US group, where more explicit techniques were employed in the US group (*p* < 0.001).

## 4. Discussion

Through exploring online stores selling disposable e-cigarettes and tweets related to disposable e-cigarettes, we characterized the popular flavors of disposable e-cigarettes and identified topics related to disposable e-cigarettes on Twitter. The top popular flavors of disposable e-cigarettes discussed on Twitter were “strawberry” and “mango”, which were also top flavors sold online. Commercial tweets dominated in all collected tweets related to disposable e-cigarettes from March to September 2020. Explicit techniques such as “product promotion” and “flavor promotion” were more frequently used than implicit techniques such as “sales contact” and “interaction”. In addition, explicit techniques were more frequently used in the US group than the non-US group. Explicit techniques exposed more disposable e-cigarette products and flavors to Twitter users (especially young people) than implicit techniques, which could increase the probability of initiation of disposable e-cigarette use, given the previous identified positive association between e-cigarette advertising exposure and e-cigarette use [11,12].

The number of relevant tweets from the US remained stable during the study period despite the legislation, which could suggest that the legislation did not lead to a switch to prmoting disposable flavored products. In 2020, 38% of Twitter users were young adults (aged between 18 and 29 years old) [13]—a population which could be susceptible to being influenced by advertising to begin a long-term nicotine addiction. However, the stable trend might not suggest the success of the legislation since the promotion of disposable e-cigarettes might have risen in other social media platforms (such as TikTok and Instagram) that were used more often by young people during the study period. In addition, the increasing popularity of disposable e-cigarettes imposes more severe environmental pollution than previous refillable cartridge-based e-cigarettes.

Few studies discussed the disposable e-cigarette flavors and topics using social media data. A recent study collected some brand and flavor information from Reddit data that mentioned disposable “pod-mods” in September and October 2019 [14]. In addition to the brands that were mentioned in their study, we collected disposable e-cigarettes brands and flavor lists from the online stores selling disposable e-cigarettes. The top flavors of disposable e-cigarettes that we collected from online stores and disposable e-cigarette-related tweets are consistent with their study, finding that “mint” and “menthol” are popular flavors. Our study also indicated that fruit flavors such as “strawberry” and “mango” in disposable e-cigarettes that appeal to youth were still promoted in the US using Twitter, which could reduce the effectiveness of the FDA flavor enforcement policy and undermine public health.

There are several limitations to our study. First, Twitter was the only social media platform used in the current study, which could not cover the whole picture of disposable e-cigarette promotions on social media. Other social media platforms that are more often used by youth, such as TikTok, Snapchat, and Instagram, or disposable e-cigarette online forums could be further explored in future studies for the disposable e-cigarette promotions. Second, the relatively small sample size of tweets related to disposable e-cigarettes on Twitter limited the power of more robust findings. Third, the relatively small proportion of youth on Twitter limited the use of Twitter data for exploring public perceptions of disposable e-cigarettes. Other social media platforms, such as TikTok, Snapchat, and Instagram, disposable e-cigarette online forums, or national surveys could be additional resources for examining public responses to disposable e-cigarettes, especially among the youth, to obtain more robust results. Fourth, the geolocation of Twitter users could only be collected if the users chose to share this information. This limited the sample sizes of these two groups. Moreover, since the brands and flavors of disposable e-cigarettes were collected manually, we could have missed some brands and flavors that lack popularity. In addition, we were unable to examine the topics in noncommercial tweets related to disposable e-cigarettes due to the small sample size. Future studies on user perceptions of disposable e-cigarettes will be conducted when more noncommercial tweets become available.

## 5. Conclusions

Fruit (strawberry, mango, and watermelon) and mint flavors were the most popular flavor categories of disposable e-cigarettes discussed on Twitter. Almost all collected tweets related to disposable e-cigarettes on Twitter were commercial tweets, with “product promotion” and “flavor promotion” as the most frequently used techniques for promoting the sale of the disposable e-cigarettes on Twitter. The results suggest that disposable e-cigarette companies and retailers took advantage of loopholes in the legislation to promote flavored disposable e-cigarettes to young people, which could compromise public health if they get addicted to e-cigarettes. Our study shows the limitations of using Twitter to study youth use of e-cigarettes, because we found that the number of tweets on disposable e-cigarettes remained stable, whilst other evidence suggests that their use grew.

## Figures and Tables

**Table 1:** Popular flavors in disposable e-cigarettes.

Flavors	Frequency from Online Stores	Flavors	Frequency from Tweets
Mango	96	Strawberry	51
Blueberry	96	Mango	44
Strawberry	89	Watermelon	42
Mint	56	Mint	35
Peach	58	Banana	31
Lemonade	56	Menthol	30
Banana	56	Cola	29
Pineapple	56	Lemon	28
Apple	55	Grape	27

**Table 2:** Topics related to disposable e-cigarettes discussed on Twitter and comparison of topics between tweets from the United States (US) and non-US countries.

Group	Topics	Total, *N* (%)	US, *N* (%)	Non-US, *N* (%)
Explicit techniques	Product promotion	634 (43.85%)	171 (52.62%)	298 (43.83%)
	Flavor promotion	220 (15.21%)	86 (26.46%)	68 (10%)
	General advertisement	106 (7.33%)	33 (10.15%)	39 (5.76%)
	Price promotion	117 (8.09%)	10 (3.08%)	62 (9.12%)
	New arrival	78 (5.39%)	12 (3.69%)	44 (6.47%)
Implicit techniques	Sales contact	113 (7.81%)	7 (2.15%)	16 (2.35%)
	Interaction	178 (12.31%)	17 (5.23%)	91 (13.38%)
	Related information	62 (4.29%)	4 (1.23%)	36 (5.29%)
	Catchphrase	42 (2.90%)	5 (1.54%)	27 (3.97%)
	Unrelated wishes	15 (1.04%)	0 (0.00%)	11 (1.62%)
	Company culture	8 (0.55%)	0 (0.00%)	4 (0.59%)
	Advocate disposable	7 (0.48%)	0 (0.00%)	4 (0.59%)
Others	Unsorted	52 (3.59%)	11 (3.38%)	35 (5.15%)

## Supplementary Materials

The following are available online at https://www.mdpi.com/1660-4601/17/24/9221/s1: Table S1. Available disposable e-cigarettes collected from online stores.
