# Supplementary material for: Promotion of Disposable Electronic Cigarette Flavors and Topics on Twitter
Source: Int J Environ Res Public Health. 2020 Dec 10;17(24):9221. doi: 10.3390/ijerph17249221 (PMC7764575; doi:10.3390/ijerph17249221)
Supplement: Supplementary file 1 [file ijerph-17-09221-s001.pdf]

Table S1. Available disposable e-cigarettes collected from online stores.

Brand Name	Product Name	Flavor
mojo	mojo disposable vape	ice pineapple, strawmelon, grape, cool melon, menthol, lemmon dessert, classic tobacco, peach, mango, strawberry, respberry, cubano
plus pods	plus bar disposable pod device	pineapple, blueberry, mango, blue raspberry, california, pineapple ice, banana ice, soul bound, strawberry banana, watermelon, lemonade, kiwi ko, sweet apple, grape, lychee ice, lush ice, cool mint, euphoria, cosmic banana, orange burst, mint, iced peach, banana, melon
stig	stig disposable vape device	tropical mango, mighty mint, dry tobacco, iced mango bomb, crisp apple, ice purple bomb, cubano, lush ice
puff	puff bar disposable pod device	orange mango guava, strawberry banana, blueberry ice, strawberry, tangerine ice, cool mint, grape, menthol, grava ice, cucumber, cool ice, café latte, O.M.G, melon ice, lush ice, blue razz, banana ice, peach ice, pomegranate, sour apple, pink lemonade, peach ice, mango, pineapple lemonade, blue razz, watermelon, lychee ice, tobacco, cool mint
	puff xtra disposable pod device	apple sweet, pineapple lemonade, naked pineapple, pink lemonade, strawberry, orange, mango lychee, mint, banana ice, cola, blueberry, grape, mango, guava ice, peach ice, gummy bear, honeydew, lush ice
	puff flow disposable pod device	aloe mango melon ice, grape apple pear, guava ice, kiwi strawberry, lychee ice, papaya banana, peach ice, strawberry banana
	puff plus disposable pod device	aloe grape, banana ice, cool mint, guava ice, lychee ice, mixed berry, peach ice, pina colada, strawberry watermelon, tangerine ice, watermelon
	puff xxl disposable pod device	lush ice, pineapple grape, mixed berries iced, papaya strawberry, aloe mango melon, watermelon cherry, cranberry lemon ice, cool mint, mango orange pomelo, banana ice
zaero	zaero disposable pod device	blueberry lemonade, peach mango, sweet orange, blue razz, tobacco, grape, menthol, banana, chocolate
fogg	fogg vape disposable pod device	watermelon, solace dragon, latte, cigar, purple berry , jewel mango, solace strawberry hard candy, mango secret sauce, solace mint, tobacco, grape
propaganda	propaganda cali bars	cookie butter, illuminati, sour apple, blue slush ice, juicy grape ice
drip more	switch mods disposable stick	green apple, pog, strawberry banana, mint, strawberry lemonade, grapefruit guava, watermelon ice, pink lemonade, blue razz, berry, melons, mango
ezzy	ezzy oval disposable pod system	pineapple twist , orange soda, lush ice, blue razz, guava ice, icy cola, vanilla custard, frozen banana, mamba, caramel popcorn, lychee ice, pina colada, berry cool, mango lychee, honeydew ice, kiwi strawberry, guava iced, lemon mint, strawberry lemonade
	ezzy super disposable device	guava ice, honeydew ice, pomegranate ice, pina colada, mango lychee
cali pods	cali pods air disposable	blueberry pomegranate, blue raspberry, lush ice, grape, mango, mango orange guava, mighty mint, orange peach, pink lemonade, strawberry, strawberry kiwi, watermelon
california grown	cali bars	honeycomb berry, sweet grapefruit ice, unicorn milk ice, mega melons ice, rainbow ice, sweet mint, mango nectar, lychee ice, watermelon, peach ice, strawberry ice, blue razz ice, blue razz, mango nectar, strawberry banana ice
wave bars	wave barsdisposable device	vanilla ice, tobacco, straw mango, S.I.C. menthol, pineapple, pina colada, peach ice, P.M.P., nana ice, mint, mango ice, lychee, lush ice, honeydew, lemon cake, gummy ring, grape, caramel popcorn, blue raz
sea pods	sea pod disposable pod device	blueberry, blueberry menthol, lush on ice, mint, mango, pineapple lemonade, pinapple lemonade, raspberry, strawberry, raspberry menthol, strawberry menthol, watermelon
	sea air disposable pod device	green apple, milky mango, guava green apple, green soda, sweet mint, kiwi red apple, mango, mint, red apple, orange sherbert, blue slush,

Brand Name	Product Name	Flavor
		strawberry watermelon banana, orange soda, peach ice, pineapple, frosted red apple, strawberry watermelon
	sea stix disposable pod device	blue slushine, apple kiwi ice, sweet strawberry, passion fruit orange guava, banana ice, fresh mint, pear ice, watermelon bubble gum, peach ice, pina colada, orange soda, pink lemonade, pomegrante lemonade punch, lush ice
pop	pop disposable vape	blue razz, iced lychee, iced pineapple, mango, banana ice, mighty mint, lush ice, mango peach pineapple, pineapple strawberry coconut, mango peach, strawberry mango, fresh tobacco, strawberry lemonade
	pop xtra disposable device	passion fruit organge mango, sour chew, chilled kiwi strawberry, chilled passion fruit, iced pineapple, custard craze, chilled blue razz, sour chew
posh	posh disposable vape	mango frost, blueberry ice, banana smoothie, OMG tropical, frozen mango, minty berry, frozen grape, lush ice, pink lemonade, cool melon, pineapple slush, cool mint
	posh plus vape	menthol ice, frosted apple, frozen strawberry, tobacco menthol, minty , minty lychee, banana smoothie, pina colada, minty melon, ice grape, cream brulee
	posh plus XL vape	frozen apple, cool grape, blue razz ice, strawnana ice, cali peach, arctic melon, frozen apple, kiwi berry ice, might mint, minty strawberry, peach iced
mr. vapor	mr. vapor disposable pod divice	grape ice, watermelon, blueberry raspberry, blueberry menthol, passion fruit orange guava, mango, peach, pineapple orange, orange mango guava, strawberry, strawberry kiwi, pink lemonade, strawberry banana, strawberry lemonade ice, cool mint, lush ice, apple ice, pineapple lemon
lava2	lava stik disposable vape pen	banana, ice grape, splash ice, summer passion, lychee, banana ice, blueberry, strawberry banana, watermelon with mint, cool mint, peach, fab tobacco, mango ice, apple with mint, chill menthol
fliq	fliq disposable vape device	blueberry, pineapple, sweet mango, pink lemonade, menthol, tobacco, fuji apple ice, pink mango ice, strawberry jubilee ice
RELX	RELX nano	white freeze, fruit tea, dark sparkle, classic tobacco
	RELX nano 2	fresh red, ludou ice, fruit tea, classic tobacco
TWST	twst disposable pod device	pink punch lemonade, arctic cool mint, iced fruit punch, berry medley lemonade, custard tobacco
epuffer	eco disposable e-cigs	tobacco, caramel mocha, menthol
	xpod mini vape	tobacco , menthol, mango
wow	wow disposable vape	tobacco, watermelon, peppermint, menthol, coffee, pina colada
ziip lab	ziip zstick disposable pod device	berry mix, blue raspberry, fresh mint, grape, iced blueberry, iced cola, iced grape, iced pina colada, iced strawberry, iced pineapple, lush ice, mango, peach, strawberry lemonade, strawberry milk, tobacco, watermelon, watermelon lemonade , banana pineapple, cappuccino, double apple, fresh mint, inced orange, iced tobacco, iced watermelon, lychee, P.O.G
nano	NANO DISPOSABLE PORTABLE POD SYSTEM	Strawberry, Pink Lemonade, Pineapple, Mango, Mango Ice, Lychee, Lush Ice, Cake, Blueberry
Econ Smoke	ST!K disposable vapes	mango, blue rasberry, peach, cubano, kiwi strawberry, green apple, pineapple, blueberry, strawberry, citrus, watermelon, lush ice, cucumber, pink lemonade, strawberry banana, berry gelato, pomegranate
	Eon Pure Distillate CBD Cartridge	blue dream, purple haze, OG Kush
	nano CBD	Mint Extract
	pre-rolled CBD	blue dream, purple haze, OG Kush
bidi	bidi stick	berry blast, jungle juice, dragon venom, kick start, lush ice, mint freeze, blazing vibe, icy mango, champion juice, fruity mango
	blazer bar	iced apple, tobacco, mixed berry, cinnablzed, menthol, mango, rainbow
blow	blow disposable vape stix	raspberry mint lemon, mango, grape ice, pineapple ice, green apple, blueberry raspberry, deeeewy, tangerine ice, fire bull, strawberry mango,

Brand Name	Product Name	Flavor
		snow, strawberry watermelon, bursted cherry, monsta fuel, orange guava passionfruit, lemon ice
hyppe bar	hyppe max vape	lush ice
	hyppe bar disposable pod device	apple ice, blueberry iceblue raz, cool melon, cola ice, frozen banana, frozen mango, grape soda, icy mint, guava iced, lemon ice, lush ice, lychee ice, lychee soda, mango lychee, orange soda, peach ice, peach soda, pear ice, pina colada, russian cream, pineapple soda, strawberry banana, strawberry ice, tropical mix
level disposable	level disposable vape	strawberry ice, peach ice, frozen banana, melon ice, mango berry, frozen mint, raging bull, cali berry, apple ice, lychee ice
Mylé	Mylé Minis disposable vape	peach ice, strawberry banana, iced blueberry, banana ice, mixed berries, raspberry watermelon, grape ice, strawberry mango ice, peach, iced lychee, pink lemonade, lemon mint, red apple, iced mint, iced quad berry, iced watermelon, sweet tobacco, iced apple mango, mango
Oro disposables	oro vape bar disposable device	strawberry ice, frozen banana, peach ice, lychee ice, lush ice, apple ice, melon ice, mango, blue razz, orange soda
shade	shade disposable pod device	blue razz, blue slushy, mango ice, melon craze, mojito, mustery, peach mango watermelon, pineapple ice, pink punch lemonade, strawberry banana
suorin	suorin air bar	banana ice, banana pineapple, blueberry ice, cherry cola, cool mint, cucumber, grape ice, hami melon ice, lychee ice, mango, O.M.G, mango strawberry, P.O.P, peach lemon, peach, pineapple ice, pink lemonade, sour apple, strawberry kiwi, strawberry lemon, strawberry watermelon, watermelon candy, watermelon ice
smash bar	smash bar vape	blue razz, blueberry ice, cool mint, O.M.G, pineapple lemonade, watermelon
fresh bar	fresh bar disposable pod device	grape, lush ice, pychee ice, mango, mint, pink lemonade, rebel
hit	hit plus disposable pod device	horizon peach, midnight mango, minty lychee, watermelon burst, strawberry sunlight
	hit disposable device	pear ice, guava iced, banana milkshake, lychee ice, blueberry, might mint, banana ice, grape, pineapple, lush ice, mango, blue raz, cherry ice
star pods	star pods plus disposable pod device	blue razz, mint, O.M.G, straw banana
breeze	breeze plus disposable pod device	banana freeze, cool mint, fizzy iced cola, green apple ice, minty berry, pineapple lemonade, pink lemonade, red apple, strawberry ice
hit stick	hit stick disposable pod device	winter apple, blueberry chill, strawberry bull, mangolicious, peach lced, mint iced
hqd	hqd cuvie disposable	blueberry, mango, orange breeze, cantaloupe, pineapple, strawberry, cheesecake, banana ice, lush ice, apple crush, ice mint, mixed fruits, grapey, strawberry banana, lychee ice, green blast, peach ice, melon ice, blue razz, tangerine ice, watermelon, bubblegum
	hqd cuvie v2 disposable	banana ice, mango ice, strawberry, peach ice, mixed fruits, ice cola, cherry guava, blueberry, ice mint, grapey, nuts tobacco, pineapple, orange soda, lush ice, lychee ice, tobacco, cherry
	hqd maxim disposable pod	blueberry, mango, grape, orange breeze, cantaloupe, mixed fruits, pineapple, strawberry, peach, watermelon, banana ice
	hqd stark disposable	mango, strawberry, grape, cantaloupe, mixed fruits, lychee ice, pineapple, lush ice, banana ice, wild peach, ice cola, ice mint
loy	loy disposable device	lush ice, peach lemonade, kiwi strawberry, strawberry, mango peach, mighty mint, pink lemonade, watermelon lemonade, strawberry mango, strawberry watermelon, ice pineapple
	loy xl disposable device	banana milkshake, kiwi strawberry, mighty mint, blue raz, mango grape, lush ice, candy, pineapple ice, strawberry watermelon
supreme	supreme cig disposable device	bubble gum, strawberry banana, lush ice, grape, apple ice, melon ice, blue razz, pineapple ice, kiwi pineapple, cool mint

Brand Name	Product Name	Flavor
jak	jak classic disposable	tobacco, menthol, vanilla, apple passion, pineapple, blackberry, blueberry, grape, watermelon, coconut, cherry, mango, wildberry, strawberry, fruit, mandarin classic, fireball, peach hookahs
smok	smok fyre disposable pod device	banana ice, blueberry, blue raz, cool mint, lush ice, lychee ice, mango, pineapple lemonade, pink lemonade, sour apple, strawberry, watermelon
dinner lady	dinner lady disposable vape device	lemon tart, blue menthol, smooth tobacco
lost vape	lost vape mana stick disposable	banana ice, blue razz, honeydew, lush ice, lychee ice, menthol, orange pop, passion fruit, peach tea, pineapple ice, purple haze, tobacco
	lost vape mana stick r disposable pod	pina colada, menthol, aloe jelly, rainbow drop, sea salt lemon drop, purple haze, lush ice, passion fruit, orange pop, pink lemonade, strawberry banana, watermelon strawberry
esco bars	esco bar disposable device	El Frio, El Papaye, El Guave
omni	omni disposable device	grape ice, mint, passion fruit, strawberry lemonade, lush ice
hyde	hyde disposable	mango ice, blue razz, cherry lemonade, honey dew punch, lush ice, pineapple ice, pink lemonade, strawmelon apple, fire & ice, krazy kustard, strawberries & cream, mango, spearmint, peach mango watermelon, strawberry banana, banana ice, sparkling orange, wild golden apple, cinnamon banana, pineapple peach mango, polar menthol, arctic menthol, glacier menthol, ice menthol, black ice menthol, bold tobacco, american tobacco, rich tobacco, gold tobacco, smooth tobacco
	hyde curve s edition disposable	krazy kustard, pink lemonade, straw melon apple, mango, mango ice, pineapple ice, peach mango watermelon, wild golden apple, cinnamon banana, strawberries & cream, blue razz, cherry lemonade, honeydew punch, lush ice, spearmint, banana ice, sparkling orange, strawberry banana, pineapple peach mango, polar menthol, ice menthol, arctic menthol, black ice menthol, glacier menthol, tobacco bold, tobacco rich, tobacco smooth, tobacco american
	hyde color edition disposable	cherry lemonade, blue razz, honeydew, peach mango watermelon, spearmint, lush ice, fire & ice, krazy kustard, pineapple ice, mango, mango ice, strawmelon apple, strawberries & cream, pink lemonade, sparkling orange, banana ice, strawberry banana, lemon crumble, pineapple peach mango, aloe grape , cinnamon banana
bolt	bolt disposable	crape ice, lush ice, lychee ice, mango ice, peach ice, melon ice cream, orange pop, red apple, strawberry peach, watermelon chew
hero	hero disposable stick	strawberry banana cream, strawberry cream, strawberry lemonade ice, blue raspberry ice, cola ice, green apple ice, lush ice, cherry lemonade, banana ice, cool mint, PPMS
mio	mio stix disposable	icy lychee, icy lush, icy mango, blue slushy, icy peach, icy watermelon, icy blue razz, icy banana, gold tobacco, icy menthol, icy strawberry milk, limon cola, cuban tobacco
moti pin	moti pin disposable	pineapple ice, tropical mango, banana frost, grape ice, blueberry parfait, spearmint, watermelon ice, mango lychee ice, strawberry ice, blueberry ice
loosie	lossie stick disposable	peach ice, lush ice, banana ice, blue raspberry, strawberry banana, strawberry ice cream, strawberry kiwi, cantaloupe ice, pink lemonade, orange mango guava, lychee strawberry, donuts, pina colada, rainbow, strawberry cheesecake
fury	fury stick disposable	mango, blue raspberry, blueberry mint, green apple, pink lemonade, strawberry, virginia tobacco, watermelon mint, banana strawberry, cola ice, lychee ice, cherry, blueberry ice
happy sticks	happy sticks disposable	strawberry lemonade, lush ice, green apple, mango, cool mint, strawberry mango, ice blueberry, cherry, joyberry, grape, watermelon strawberry, banana
fog x vapor	fog x disposable device	strawberry wafer, mango ice, pineapple ice, white grape ice

Brand Name	Product Name	Flavor
BLVK Unicorn	blvk unicorn uniciig v2 disposable device	spearmint, red orange ice, mint chocolate, mango ice, cuban cigar, cantaloupe ice
swft plus	swft plus disposable device	banana ice, cola ice, grape, lush ice, latte ice, peach ice
side bae	side bae 50 disposable device	georgia peach, iced honeydew, fresh pineapple, iced blue raspberry lemonade, strawberry acai, watermelon candy, iced lychee, iced wintergreen, blue raspberry lemonade, passion fruit
barz	barz disposable device	banana ice, lychee ice, mango guava, strawberry banana, melon ice, blue razz, lush ice, cool mint, cola ice, grape ice, lemon grape fruit
hale puff	hale puff disposable device	sour apple, frozen mango, frozen banana, hawaiian burst, frozen guava, frozen pomegranate
helix bar	helix bar disposable device	cake, mango ice, apple ice, tobacco, watermelon ice, blue raspberry, blue raspberry ice, mint, strawberry, strawberry ice
drag monster	drag monster disposable	strawberry banana, lush ice, strawberry lemonade, banana bread, mixed berry, orange soda, banana custard ice
loy	loy xl disposable device	mango grape, lush ice, candy, pineapple ice, strawberry mango, strawberry watermelon, banana milkshake, kiwi strawberry, mighty mint, blue raz
	loy disposable device	mango peach, pink lemonade, lush ice, iced lychee, blue razz, strawberry, watermelon lemonade, strawberry lemonade, peach lemonade
tesla cigs	tesla cigs munch disposables	strawberry ice cream, pink lemonade, milk cookie, lime, vanilla kustard, triple berries lemon, triple berries, strawberry cheesecake, tiramisu, mango lychee, lychee ice, lemon mint, lemon tart, energy drink
ryse	ryse disposable vape	banana ice, blueberry ice, grape, cool mint, lush ice, lychee ice, mango, melon ice, menthol, peach ice, pineapple lemonade, lemonade, pink lemonade, tangerine, strawberry, watermelon
pod king	pod king disposable	blueberry, strawberry mango, mango peach pineapple, white peach raspberry, orange soda, strawberry kiwi, strawberry watermelon, watermelon lemonade, strawberry banana, blueberry ice, lush ice, melon ice, banana ice
	pod king plus disposable device	strawberry watermelon gum, strawberry ice, guava ice, peach ice, orange soda, honeydew watermelon ice, peach orange, mango peach kiwi ice
	pod king maxx disposable vape stick	cherry lime soda, tiger blood, strawberry watermelon gum, lush ice, iced blue razz, mighty mint, apple peach pear, grape apple iced, strawberry lemonade, banana milk, orange pineapple, blueberry pomegranate iced, lemon iced, strawberry dragon fruit peach, blood apple ice, strawberry peach lemonade iced, strawberry cheesecake, mango cake, chocolate mint
nitro	nitro's cold brew solos disposable	apple, chocolate, macchiato, mango
viigo	viigo disposable device	coffee tobacco, mint chewing gum, mixed fruit, ice apple
hitt	hitt disposable vape pen	strawberry, fresh mint, pog, lychee ice, mango, banana ice, punched iced, grape jam, guava, smooth tobacco, peach ice, pineapple dream, lemonade ice, blueberry pomegranate, watermelon, kiwi berry, lush ice, melon ice, milkshake, very berry
Mr Freeze	Mr freeze vape disposable	mango frost, mango, watermelon frost, apple frost, blue raspberry frost, strawberry watermelon frost, mighty mint, strawberry banana frost, peach frost, pure ice, berry frost, tobacco, strawberry lemonade, strawberry lemonade frost, banana frost
lemon twist	lemon twist disposable vape	iced fruit punch, iced madness, iced pink punch, pink punch lemonade
skol	skol uno disposable pod device	cushman, cool mint, strawberry yogurt, lush ice, iced blueberry, strawberry lemonade, iced lychee, mango iced, grape, pink lemonade, watermelon ice, pina colada
ezee	ezee stick disposable puffs	grape, peach, watermelon, cool mint, menthol, lychee ice, peach ice, strawberry, strawberry kiwi, pineapple ice, sour apple, strawberry banana, pineapple, banana ice, mango, blueberry raz ice, tobacco

Brand Name	Product Name	Flavor
fizz vapor	fizz disposable vape pen	cactus jackfruit mandarin, pineapple, banana ice, lush ice, strawberry kiwi, pomegranate ice, mango peach guava, cucumber, peach, strawberry, strawberry banana, blueberry pomegranate, blueberry, mint chewing gum, pink lemonade, blueberry raspberry, lychee ice, green apple, café latte, mango
lit	lit disposable vape pen	mango, baked, strawmelon, iced blue slushine, menthol
just mango	just mango disposable pod device	fresh orange kiwi ice, fresh pineapple guava, fresh strawberry coconut ice, fresh sweet mango ice, fresh sweet peach ice, orange kiwi, pineapple guava, strawberry coconut, sweet mango, sweet peach
myst	myst disposable device	blueberry, pink lemonade, watermelon, cool mint, mango, orange, pineapple
bluumlab	bluumlab disposable CBD infused pod	berry, mango, tobacco, mint
natures tru	natures tru disposable pod device	dark OG kush, lush ice, blueberry bliss, mango tango, strawberry slam
MR FOG	mr fog disposable pod	grape, sour apple, watermelon, mint, strawberry, pina colada, pink lemonade, bubble gum, blueberry mango, blueberry, mango
4x pods	4x pods disposable pen	blue raspberry, kiwi strawberry, peach madness, ripe mango
axe	axe disposable vape pen	blueberry, lush ice, mango, menthol, pineapple crash, pink lemonade
candy bar	candy bar xl disposable vape	banana ice, blueberry, froopy, tropical mango, mint, strawberry ice, crisp apple, blpeach ice, banana smoothie, lychee ice, watermelon ice
	candy bar max disposable vape	banana shake, tiramisu, red apple ice, guava ice, pineapple tangerine, lychee ice, mango ice, blueberry ice, cotton candy, strawberry milk
cloud puff	cloud puff pro disposable vape	peach pineapple, frosted apple, blue raspberry, strawberry kiwi, lemon ice, peach ice, honeydew mint, banana ice, pink lemonade, passion fruit, strawberry melon apple
crushed	crushed xl disposable vape device	berry watermelon gum, iced frosted apple, iced balck cherry, melon berry crush, iced berry banana, pineapple crush, blue raz, iced grape crush, iced mint, passion fruit ice, blueberry lemonade, blueberry crush, iced banana crush, lush ice
	crushed air disposable vape	watermelon candy, mighty mint, sour apple, mango guava ice, lush ice, guava peach ice, blueberry crushed, peach crushed
drip	drip disposable vape pen	red apple ice, melon chews, mint, mango ice, cotton clouds, lychee ice, pina colada ice, lush ice, peach ice, strawberry ice, apple crush, melon peach rings
dspo	dspo disposable vape pen	pebbles iced, pineapple slushine, apple orchard iced
	dspo x disposable vape pen	pineapple coconut ice, strawberry banana iced, iced apple
eleaf	eleaf disposable vape	lychee ice, watermelon ice
exhale	exhale disposable vape pen	grape escape, orange mint, peachy keen, mango tango, watermelon chill, strawberry watermelon, dutch apple, banana bash
	exhale plus disposable vape pen	blue raspberry, banana ice, strawberry banana, mint, lychee ice, mango, georgia peach, tropical escape, pink lemonade
fruyt pods	fruyt stik disposable pen	blueberry blast, juicy watermelon, lush ice, mango, mixed berries, sour apple, strawberry delight, strawberry delight, strawberry lemonade, tropical pineapple
iplay	iplay vino disposable vape device	sweet melon, snow king, red wine, pineapple lemon, grape soda, peach ice, orange, mixed berries, mango ice, banana ice
just rock	just rock disposable vape pen	tropical balst, summer watermelon'arctic mint, mango dush, havaiian rock, twisted lush, wild beach, sunny crazy, blue razz, jungle rock
legit	legit disposable vape pen	banana ice, georgia peach, double apple, strawberry ice, mango ice, blueberry ice
mood	mood disposable vape device	blueberry ice, lush ice, lychee grape, mango ice, orange ice
phantom	phantom disposable device	lush ice, mango splash, mango peach, melonberry ice, pineapple ice, mango ice, pink lemonade, iced mint, strawberry ice, blueberry ice
pixxi pro	pixxi pro disposable vape pen	georgia peach iced, watermelonade, raging bull, frosted gummmy bear, frozen green apple, blue razz, iced out watermelon, mint, iced out strawberry peach

Brand Name	Product Name	Flavor
sixt	sixt disposable pod device	green apple, O.M.G., orange kiwi, strawberry kiwi, mango, pineapple, P.O.G., pink lemonade, café latte, watermelon ice, blue raz, grape ice, no bull
smoq	smoq disposable vape	OMG, cool mint, mango, blueberry ice, pineapple, pink lemonade, lush ice, lychee ice
vaporlax	vaporlax disposable vape	banana ice, pink lemonade, strawberry cream, lush ice, orange soda, cool mint, tropical punch, blue raz, mango ice, ice cola, pineapple lemonade, peach mixed, crush grape
vozol	vozol D1 disposable pod device	refreshing mint, iced mango, yummy blast, greem apple, brain freeze, merry berries, iced summer, hawaiian pog, very cool, lava flow, american tobacco
zalt	zalt disposable devices	cool mint, blue raspberry, strawberry watermelon menthol, grape, pina colada, mango, tobacco, berry lemonade, mango ice, green apple candy, passion fruit orange guava, blueberry mint
airis	airis xl disposable device	blueberry ice, banana ice, blue razz, cool mint, kiwi strawberry, pink lemonade, lush ice, strawberry banana, strawberry watermelon, melon ice, pineapple ice, mango ice
	airis puff disposable kit	strawberry watermelon, pina colada, guava ice, cool mint, mixed berries, peach ice, watermelon
face changer	face changer disposable pod device	blue raspberry, blueberry ice, banana ice, peach ice, lush ice, strawberry watermelon bubblegum, strawberry banana, strawberry kiwi, gummy bear, rainbow candy
leap vapor	leap vapor leap go disposbale vape	citrus menthol, fresh mango, mint, smooth tobacco
lush vapor	ultra	bananaberry dragonfruit,blackberry strawberry, blue razz lemonade, chilled berry lychee, chilled passion fruit, chilled pink lemon, honeydew straw apple, kiwi strawberry, mighty mint, tropical blue razz
	lush disposable	blue razz lemonade,vreme, iced berry lychee, iced passion fruit, iced pineapple, iced strawberry lemonade, lush ice, mango lychee, mega menthol, orange soda, passion fruit strawberry , peach lemonade
killa fruit	killa fuits dispsoable	blueberry ice, cool mint, kiwi strawberry on ice, lush ice, lychee berry ice, mango, red apple peach
x2o	rush disposable device	razzletaz, mango, caribbean punch, moring mocha, menthol, tobacco, strawberry cream, apple
blu	blu disposables e-cigs	magnificant menthol, cherry crush, classic tobacco
fuma	fuma pods posh disposable	pink lemonade, pine slush, mango frost, lush ice, frozen grape, cool melon, blue b ice, cool mint, cotton candy
